# A Novel Multimodal Treatment Method and Pilot Feasibility Study for Vaginismus: Initial Experience With the Combination of Sacral Erector Spinae Plane Block and Progressive Dilatation

**DOI:** 10.7759/cureus.10846

**Published:** 2020-10-08

**Authors:** Emsal Pinar Topdagi Yilmaz, Elif Oral Ahiskalioglu, Ali Ahiskalioglu, Serkan Tulgar, Muhammed E Aydin, Yakup Kumtepe

**Affiliations:** 1 Obstetrics and Gynecology, Ataturk University School of Medicine, Erzurum, TUR; 2 Anesthesiology and Reanimation, Ataturk University School of Medicine, Erzurum, TUR; 3 Clinical Research, Development and Design Application and Research Center, Ataturk University School of Medicine, Erzurum, TUR; 4 Anesthesiology, Maltepe University Faculty of Medicine, Istanbul, TUR; 5 Clinical Research, Development and Design Application and Research Center, Ataturk University, Erzurum, TUR

**Keywords:** vaginismus, erector spinae plane block, sacral, ultrasound, multimodal treatment, progressive vaginal dilatation

## Abstract

Background

Genito-pelvic pain/penetration disorder, commonly referred to as vaginismus, is a relatively common condition in women of childbearing age and has physical and psychological aspects. Various cognitive and behavioral therapies, dilatators, botulinum injections, and so on have been tried in the treatment. We hypothesize that the combination of sacral erector spinae plane (ESP) block and progressive dilatation treatment increases treatment quality.

Methods

We performed the sacral ESP block and progressive dilatation, which we added to multimodal treatment for resistant vaginismus cases. After the procedure, all patients were followed up during one menstrual cycle. They were recommended to have sexual intercourse on the day of the procedure.

Results

In 15 of our treatment-resistant cases, when we added the sacral ESP block, successful penetration after the first block was 73%. Pregnancy occurred in eight patients after the initial one-month follow-up. Four of the 15 patients needed a second block.

Conclusions

The sacral ESP block added to the multimodal treatment protocol significantly improves treatment quality.

## Introduction

Vaginismus is a state that is seen in about 1% to 7% of women in the reproductive age group; its cause has still not been fully clarified [1]. Its incidence is unclear because patients often do not share this with health practitioners and see it as a failure. The fact that there are serious differences in its prevalence between countries suggests that this situation stems from their attitudes regarding both cultural and religious beliefs [2]. Importantly. the facts that it has a high rate of incidence in Middle Eastern countries, that virginity is blessed, that female sexuality is suppressed, and that sexuality is perceived as immorality or crime constitute the basis of cultural reasons for the difference between societies [2]. This penetration disorder, which is known as the most common cause of failed marriages, is thought to be an involuntary and uncontrolled reflex behavior. It is still unclear whether the patient really feels pain at the time of sexual intercourse or if it is due to an expected feeling of pain. The result is a vicious circle between avoidance, contraction, and pain in the pelvic muscles. It is defined as the woman's intermittent or continuous interruption of the penetration of the penis, finger, or other objects, even though she wants it to be realized. In the current approach, according to the Diagnostic and Statistical Manual of Mental Disorders (DSM 5), problems related to sexual intercourse in women are summarized. All of the clinical presentations that include pain during sexual intercourse or vaginal penetration, anxiety, fear of intercourse or anxiety, excessive contraction of pelvic floor muscles caused by anxiety-fear are collected under the title of "Genito-Pelvic Pain/Penetration Disorder" [3].

In addition to the physical aspect, various methods, such as several cognitive and behavioral therapies, relaxation of spasm with dilators, or providing vaginal desensitization by different relaxation methods, botulinum toxin, local anesthetic (LA) agents, or physiotherapy of pelvic muscles have been tried [4-5]. However, since the effectiveness of all these interventions varies individually, no standard approach to treatment has been proposed.

Sometimes, invasive methods are used in the treatment of vaginismus/genito-pelvic pain, but to date, the use of ultrasound-guided interventions called interfacial plan blocks has not been reported. The purpose of interfacial plan blocks is to utilize the effects of LA on the sensory-motor system by way of potential gaps and pathways between the fascia without direct needling-intervention/contact with the target nerve or plexus. The LA agent reaches the target nerve, possibly through the fascia/foramina. The most recent and popular one among these methods applied to the accompaniment of ultrasound is the erector spinae plane block (ESPB). ESPB was defined for the first time in 2016 by Forero et al. for the treatment of thoracic neuropathic pain [6]. Initially applied for the treatment of chronic pain from the thoracic level alone, ESPB has been reported to be administered from different thoracic levels, including postoperative pain and other acute pain conditions [7]. Later, it was identified in lumbar and sacral ESPB, and its uses for many different indications were reported [8-10]. Although sacral ESPB was initially a hypothesized technique to block only the posterior branches of the sacral nerves, it can also block the lumbosacral plexus and especially the sacral spinal nerves (S2-4) when applied at high volumes [11].

In this study, we aimed to present 15 resistant vaginismus successfully treated with a combination of sacral ESP block and progressive dilatation.

## Materials and methods

After local ethics committee approval, 15 patients were included in the study. Written informed consent from all the patients was obtained. Patients who "described sexual intercourse as impossible due to excessive pain and whose previous treatments failed" were included in this study. Lamont grade was used to grade the severity of vaginismus to improve the treatment plan and provide more appropriate support [12].

Patients who had American Society of Anesthesiologists (ASA) III and/or more risk due to comorbidity, used multiple medications, or had suffered from respiratory, cardiac, neurologic, or neurological problems were excluded from the study. All patients were given detailed information regarding the block and dilatation procedure, including potential complications. All procedures were performed by the same anesthesiologist and gynecologist.

Since the sacral ESP block is a relatively tolerable procedure, no sedative or opioid analgesic was administered during the procedure. The treatment of post-block vaginal muscles continued with digital examination using 2% xylocaine gel. Progressive dilatation without sedoanalgesia was applied to all patients 30 minutes after the block application. A multimodal approach, such as post-procedure counseling, support, and follow-up, continued in all patients. After written, informed consent was obtained from all patients, the sacral ESPB and dilatation procedures were performed. All patients were given their personal access numbers and followed up after the procedure and they were kept in touch. No information was given about the duration of the block after the sacral ESPB procedure.

Application of sacral ESP

**Figure 1:**
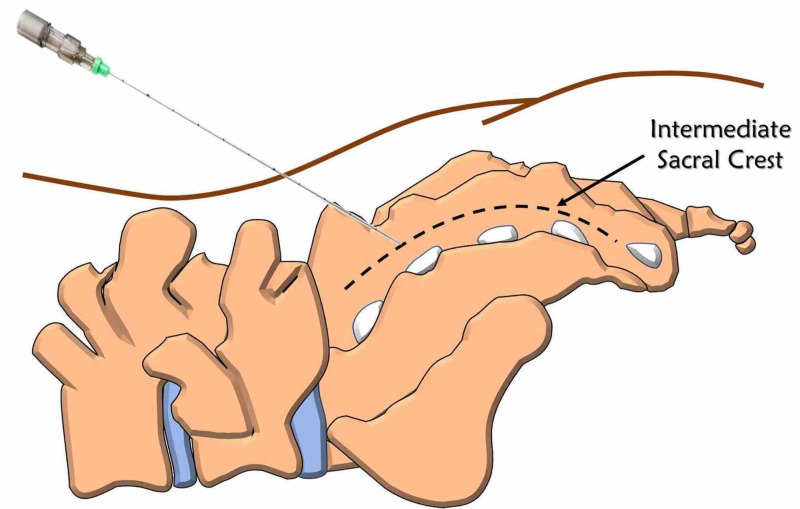
Basic illustration of sacral erector spinae plane block Image created by the authors of the current article

**Figure 2:**
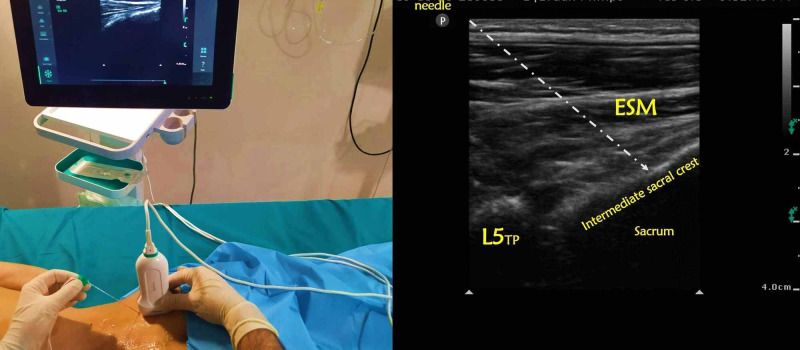
Patient and ultrasound setup for sacral erector spinae plane block; sonographic anatomy of the block L5: lumbar 5th vertebrae; ESM: erector spinae muscle

All patients were placed in the prone position. The L5 transverse process, sacrum, and S1 intermediate sacral crest were visualized by placing the curvilinear ultrasound probe in the parasagittal plane. The transducer was converted to the transverse plane, and the site was confirmed and brought to the sagittal plane. The needle was directed towards the S1 level by the craniocaudal in-plane or out-of-plane approach. The solution, including 20 ml of 0.250% bupivacaine + 8 mg dexamethasone (the dose we use in sacral ESPBs for chronic pain in our clinic), was applied between the erector spinae muscle and the intermediate sacral crest. Cranio-caudal spread of the drug was confirmed by observing that the muscle was elevated above the bone. The same procedure was repeated on the other side (Figures 1-2).

Progressive dilatation

These patients who could not tolerate the dilator in previous trials were applied the progressive dilatation process in the lithotomy position, using a plastic dilator with a circumference of 2.5 inches in the first hour after the block. The process was continued with an oversized dilator according to the patient's state of toleration. Each dilator remained one hour at an average of in the patient. The process was terminated using a dilator with a maximum circumference of 6 inches. Intercourse was recommended to the patients after application. Our most significant advantage was that patients experienced dilatation painlessly, relief in the pelvic floor, but felt dilators in the meantime.

## Results

According to Lamont-Pacik grade staging, six patients were evaluated as Stage 3, six patients were Grade 4 and three patients were Stage 5 vaginismus. The demographic data of the patients are shown in Table 1.

**Table 1:** Demographic and procedural details of study patients

		Case 1	Case 2	Case 3	Case 4	Case 5	Case 6	Case 7	Case 8	Case 9	Case 10	Case 11	Case 12	Case 13	Case 14	Case 15
Age (years)		23	27	21	29	26	30	27	32	29	32	25	23	29	23	30
Partner Age (years)		28	34	26	31	30	36	29	33	34	38	25	27	30	26	31
Living with partner (yes / no)		yes	yes	yes	yes	yes	yes	yes	yes	yes	yes	yes	yes	yes	yes	yes
Family history (yes / no)		no	no	no	yes	no	no	no	no	no	no	yes	no	no	no	no
Family type (Joint / Nuclear)		joint	joint	joint	nuclear	joint	joint	joint	joint	joint	nuclear	nuclear	joint	joint	nuclear	nuclear
Duration of marriage (month)		12	9	24	36	15	18	40	46	44	12	14	20	10	22	16
Family problems (yes / no)		no	yes	no	no	no	no	no	yes	no	no	yes	no	no	no	no
Marital problems (yes / no)		no	no	yes	no	no	no	no	yes	no	no	no	yes	no	no	no
Level of Education																
	Secondary School					+							+			
	Higher education				+		+	+	+	+				+	+	
	University Education	+	+	+							+	+				+
Lamont –Pacik grade (1/2/3/4/5)		3	3	5	4	4	4	3	5	5	4	3	4	3	3	4
Physical Therapy (yes / no)		yes	yes	yes	yes	yes	yes	yes	yes	yes	yes	yes	yes	yes	yes	yes
Successful penetration after first block (yes / no)		yes	no	no	yes	yes	yes	yes	yes	yes	no	no	yes	yes	yes	yes
Consumption of second block (yes / no)		no	yes	yes	no	no	no	no	no	no	yes	yes	no	no	no	no
Pregnancy after 1^st^ month follow-up (yes / no)		yes	yes	no	no	yes	no	no	no	no	yes	yes	no	yes	yes	yes

Fifteen patients included in the case series did not encounter any problems during both sacral ESP and dilatation procedures. After the procedure, all patients were followed up during one menstrual cycle. They were recommended to have sexual intercourse on the day of the procedure. Pregnancy occurred in eight patients after the initial one-month follow-up. The other 11 patients successfully experienced sexual intercourse. Eleven of the 15 patients whom we included in the study experienced intercourse within the first three hours after the application.

However, four patients needed sacral ESP again the next day, but they could not have sexual intercourse, although they could tolerate the 6-inch dilator in the first session. Three of four patients experienced intercourse after the second sacral ESPB procedure.

## Discussion

The success rate of our treatment plan, including the uses of sacral esp and progressive vaginal dilatation, is 73% after the first block. With sacral ESPB, it seems to be adequate to both minimize pain and prevent vaginal spasm to a certain extent and then provide enlargement using vaginal dilators and desensitize the woman against penetration. It was shown that painless penetration is possible without sedating patients and sacral ESPB; the effect of this lasts relatively long and enabled them to experience intercourse without pain.

The spasm and excessive pain that the patients experienced due to the fear of vaginal penetration at the level of phobia were stopped with a multimodal approach, including dilatators, physical therapy, psychotherapy, and sacral ESPB. A second injection was required with only four patients. By nature, this method treats physical vaginal spasm. Assuming that our primary goal is to provide sexual intercourse, we presume that with this treatment approach, whose first step is to provide analgesia, as the patient is conscious and then progress to vaginal dilators, to show the possibility of a painless relationship to the woman and make her realize that it is in the normal anatomy [13]. Regardless of the treatment program used, post-procedure counseling, support, and follow-up are crucial for success. We believe that patients having close personal relationships with us and being accessible at all times is beneficial for achieving success. However, disorders such as post-procedure anorgasmia, low libido, deception, erectile dysfunction, and hostility to the opposite sex were not followed.

Many clinicians acknowledge that the enlargement program is useful in overcoming fear and penetration anxiety as well as the physical side of vaginismus [3]. Regardless of the treatment plan, dilatation therapy is an essential part of the process, but studies have shown that more than half of the patients cannot continue treatment [14]. We believe that sacral ESP is the cornerstone of our treatment program. Establishing analgesia with a different method than the vaginal approach increased the patients' motivation in deciding to start treatment. During the dilatation process, the patient was informed about the possibility of penetration in a conscious state but without pain and loss of sensation. The initial painless experience after the block may have increased the confidence for subsequent attempts.

To date, many methods have been tried to create a useful treatment model in vaginismus. Among these, the most frequently used may be counted as vaginal dilators, lubricants, sexual intercourse counseling, physical therapy, cognitive-behavioral therapy, and hypnotherapy. The success of botulinum injection was first described in 1997 in the case of secondary vaginismus [3]. Abbott et al. found that botulinum toxin, a toxin to which they injected bulbospongiosus was significantly successful in the placebo group. And they did not detect any need for additional injections in the same patient group at eight and 14 months of follow-up [15]. In their study on 23 patients, Ghazizadeh and Nikzad et al. found botulinum toxin to be 75% successful in the 12-month follow-up [16]. Regarding the methods in which botox was evaluated, that preoperative midazolam and fentanyl injections were used in severe vaginismus cases that were observed in the studies. However, in cases where even vaginal injections are not possible, the superiority of sacral ESP block administration may become evident. Undoubtedly, there is a need for studies in which the efficacy of combinations where conventional techniques and sacral ESPB are united or different modalities are compared.

When the pelvic region and innervation are evaluated anatomically, two important nerves appear to be significant. The first one, which is the levator ani nerve originates from S3, S4, and/or S5 and provides innervation of both the coccygeus muscle and the levator ani muscle complex. The second neuronal structure, the pudendal nerve, provides urethral and anal sphincters, as well as deep and superficial perineal muscles and sensory innervation to the external genitalia. The pudendal nerve originates from the sacral nerve trunks from S2 to S4 (which makes the most significant contribution with S3). These nerves should be targeted for the relaxation of pelvic muscles [17].

In the report where sacral ESPB was defined for the first time, with the LA applied between the intermediate sacral crest on the sacrum dorsal surface and the erector spina muscle (most of which is formed by multifidus), it was hypothesized that the posterior branches of sacral nerves could be blocked and that in a patient on which pilonidal sinus surgery was applied, sacral (S-2) ESPB applied with bilateral mL LA, 20 each, clinically provided sensory block in the posterior branches of L5, S1-2-3 nerves of ESPB clinically [9]. Later, their uses were reported in chronic pain conditions associated with dorsal branches, such as cluneal nerve entrapments of sacral ESPB in lumbosacral radiculopathies [11], and as part of multimodal analgesia in hip and sacrum fracture surgeries [18-19]. Moreover, another indication for sacral ESPB is gender reassignment surgery [20].

Clinical applications and reports indicate that LA is moving from the point of application to cephalic and caudal, but there is no data-clinical observation of LA's progression through the sacral foramina into the anterior sacrum and especially blocking the S2-S4 sacral nerves. Also, no clinical observations were reported indicating that LA may progress towards the caudal through the dorsal or ventral face of the sacrum and reach the pudendal nerve. However, in our hypothesis, sacral ESPB needs to reach the root of the S2-4 sacral nerves or the pudendal nerve in order to be effective in the treatment of vaginismus so that it can provide relief to the pelvic floor muscles. We think that it may not be enough that it only reaches the pudendal nerve because the deep pelvic floor muscles' (pubococcygeus, iliococcygeus, and coccygeus) motor innervation comes directly from the sacral nerves [21]. This complex anatomy may, unfortunately, complicate treatment in vaginismus.

Considering the studies that report that the S2-S4 nerve block can be used to treat pudendal neuralgia with the transsacral approach [17], instead of passing the sacral foramina, we hypothesized that we could provide similar effects by giving LA adjacent to the intermediate sacral crest in the medial of the sacral foramen. And we explain the possible mechanism of action of sacral ESPB in both cases with craniocaudal and ventral spread. Anatomic/radiological studies are needed on this issue.

Among the invasive complementary treatment methods available, sacral ESPB is a method that will come to the fore with its ease of application, by being relatively less painful than other procedures, its ease of application anywhere under simple monitoring (of course, provided that adequate precautions are taken against cases like local anesthesia toxicity) and because it causes less anxiety due to the distance of its point of injection from the vagina. However, we should not forget that this new indication, which we determined for this recently described technique, should be verified with extensive anatomical and clinical studies. Although one of the reported side effects of ESBP is the motor block; the motor block did not develop in any of our patients.

The multimodal approach we formed is promising because of the short time needed for success, the fact that patients do not wear out during the process, and that physiological and psychological aspects can be treated together. In patients with sacral ESPB, relaxation and sensory blockage without complete motor block is essential for treatment. As with ESPBs applied in other regions, in sacral ESPB, there is LA and steroid dissemination with possible pathways between fascias, and even if the concentration to provide motor block is not reached, a contribution to the treatment is provided by reaching the concentrations that will ensure a decrease in sensory block or tonus. A sensory block was observed in the S2-S4 dermatomes in all patients.

## Conclusions

In conclusion, our findings showed that the application of sacral erector spinae plane block added to multimodal therapy in advanced vaginismus patients increases the patients' compliance with the treatment and the treatment's chance of success. Furthermore, randomized clinical studies are required to determine the generalizability of our findings.
